# Meridional ocular magnification after cataract surgery with toric and non-toric intraocular lenses

**DOI:** 10.1007/s00417-022-05740-4

**Published:** 2022-07-01

**Authors:** Achim Langenbucher, Peter Hoffmann, Alan Cayless, Jascha Wendelstein, Matthias Bolz, Nóra Szentmáry

**Affiliations:** 1 Department of Experimental Ophthalmology, Saarland University, Kirrberger Str 100 Bldg. 22, 66424 Homburg, Saar Germany; 2 Augen- Und Laserklinik Castrop-Rauxel, Castrop-Rauxel, Germany; 3 School of Physical Sciences, The Open University, Milton Keynes, UK; 4 Department of Ophthalmology, Johannes Kepler University Linz, Linz, Austria; 5 Dr. Rolf M. Schwiete Center for Limbal Stem Cell and Aniridia Research, Saarland University, Homburg, Saar Germany; 6 Department of Ophthalmology, Semmelweis-University, Mária u. 39, 1085 Budapest, Hungary

**Keywords:** Ocular magnification, Image distortion, Aniseikonia, 4 × 4 matrix calculation, Paraxial optics, Vergence formula

## Abstract

**Background:**

Overall ocular magnification (OOM) and meridional ocular magnification (MOM) with consequent image distortions have been widely ignored in modern cataract surgery. The purpose of this study was to investigate OOM and MOM in a general situation with an astigmatic refracting surface.

**Methods:**

From a large dataset containing biometric measurements (IOLMaster 700) of both eyes of 9734 patients prior to cataract surgery, the equivalent (P_IOL_eq) and cylindric power (P_IOL_cyl) were derived for the HofferQ, Haigis, and Castrop formulae for emmetropia. Based on the pseudophakic eye model, OOM and MOM were extracted using 4 × 4 matrix algebra for the corrected eye (with P_IOL_eq/P_IOL_cyl (scenario 1) or with P_IOL_eq and spectacle correction of the residual refractive cylinder (scenario 2) or with P_IOL_eq remaining the residual uncorrected refractive cylinder (blurry image) (scenario 3)). In each case, the relative image distortion of MOM/OOM was calculated in %.

**Results:**

On average, P_IOL_eq/P_IOL_cyl was 20.73 ± 4.50 dpt/1.39 ± 1.09 dpt for HofferQ, 20.75 ± 4.23 dpt/1.29 ± 1.01 dpt for Haigis, and 20.63 ± 4.31 dpt/1.26 ± 0.98 dpt for Castrop formulae. Cylindric refraction for scenario 2 was 0.91 ± 0.70 dpt, 0.89 ± 0.69 dpt, and 0.89 ± 0.69 dpt, respectively. OOM/MOM (× 1000) was 16.56 ± 1.20/0.08 ± 0.07, 16.56 ± 1.20/0.18 ± 0.14, and 16.56 ± 1.20/0.08 ± 0.07 mm/mrad with HofferQ; 16.64 ± 1.16/0.07 ± 0.06, 16.64 ± 1.16/0.18 ± 0.14, and 16.64 ± 1.16/0.07 ± 0.06 mm/mrad with Haigis; and 16.72 ± 1.18/0.07 ± 0.05, 16.72 ± 1.18/0.18 ± 0.14, and 16.72 ± 1.18/0.07 ± 0.05 mm/mrad with Castrop formulae. Mean/95% quantile relative image distortion was 0.49/1.23%, 0.41/1.05%, and 0.40/0.98% for scenarios 1 and 3 and 1.09/2.71%, 1.07/2.66%, and 1.06/2.64% for scenario 2 with HofferQ, Haigis, and Castrop formulae.

**Conclusion:**

Matrix representation of the pseudophakic eye allows for a simple and straightforward prediction of OOM and MOM of the pseudophakic eye after cataract surgery. OOM and MOM could be used for estimating monocular image distortions, or differences in overall or meridional magnifications between eyes.


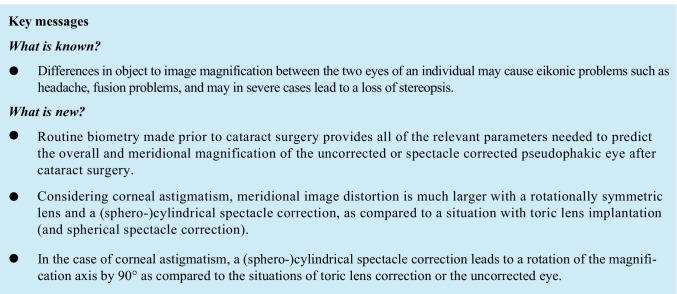


## Introduction

In modern cataract surgery, the retinal image size disparity is widely ignored [1–3]. The main reason for image size disparities is a mismatch between the biometric data of both eyes which include axial length, corneal curvature or power, or the axial position of the intraocular lens (IOL) implant. In general, intra-patient image size disparities are referred to as aniseikonia, but we have to strictly differentiate between a mismatch of overall retinal image sizes and a mismatch of retinal image size in different meridians which could be observed monocularly or binocularly [1, 4].

**Fig. 1:**
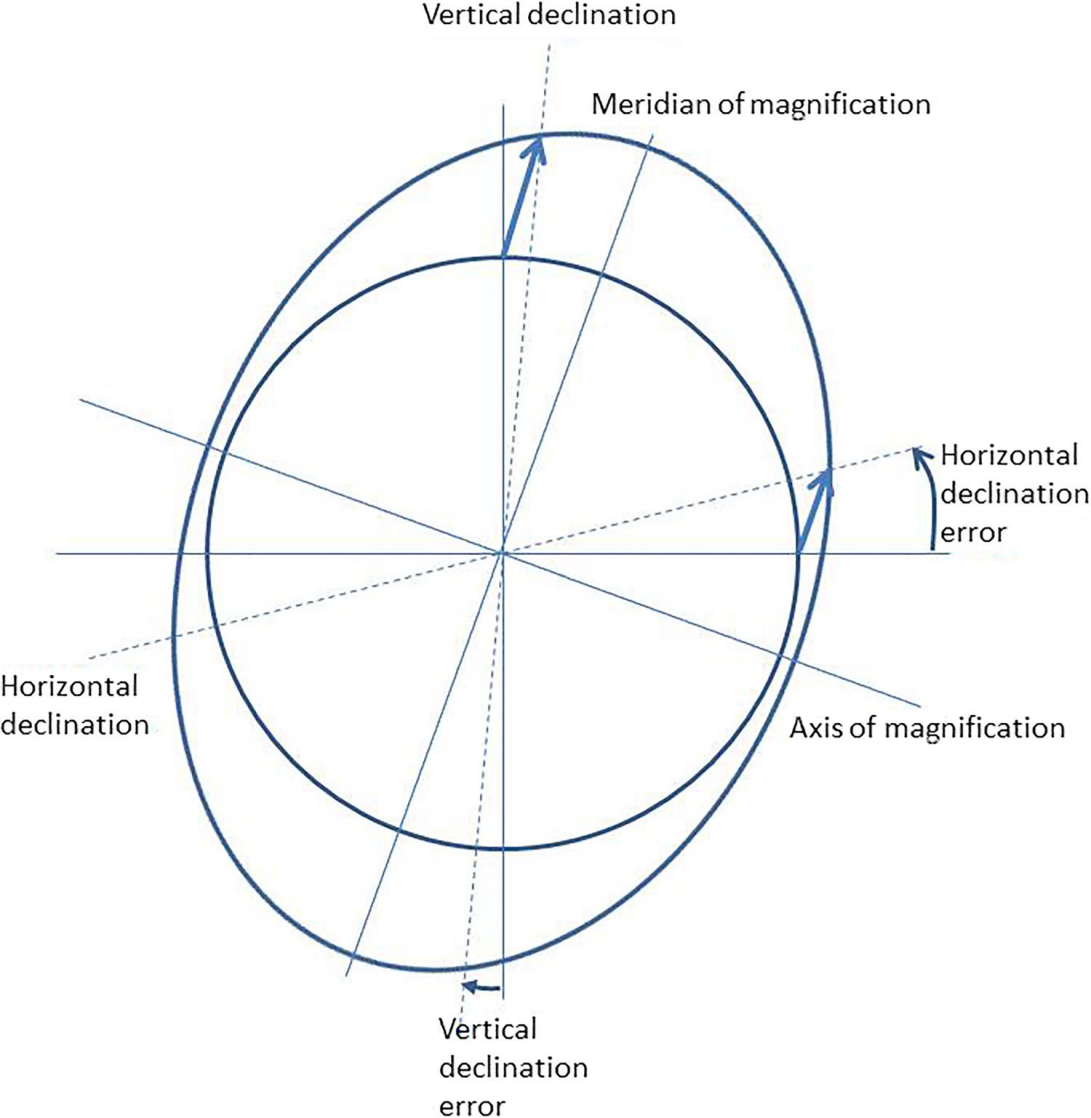
Situation of ocular magnification with spherocylindric surfaces. A circle in the object plane is distorted to an ellipse in the image plane. The meridian with the largest magnification is called the magnification meridian or meridian of magnification, whereas the meridian with the smallest magnification refers to the magnification axis or axis of magnification. In situations with 2 spherocylindric elements where one element corrects the astigmatism of the other element (e.g. corneal astigmatism fully corrected by a toric lens or spherocylindric spectacles), the magnification meridian coincides with the meridian of highest power of the first spherocylindric element

Ocular magnification (OM) refers to the ratio of retinal image size to the corresponding object size for objects at finite distances, and to the ratio of retinal image size to the incident ray angle (in radians) for objects at far distances [1]. With rotationally symmetric surfaces in the eye, we simply deal with overall ocular magnification (OOM) without variation of meridional ocular magnification (MOM), whereas for eyes with at least one toric surface, OM varies between meridians and the disparity between MEM in the magnification meridian and the magnification axis (DOM) causes image distortion. In simple cases where we deal with spherocylindric surfaces, a circle in the object space is translated to an ellipse in the image space, and the meridian of magnification refers to the semimajor axis having the largest MOM and the magnification axis refers to the semiminor axis of the ellipse where MOM is the smallest (Fig. 1).

Modern optical biometers and advanced IOL power prediction strategies can significantly reduce the prediction error of postoperative refraction and today in a highly selected cataract population 70 to 90% of eyes end up with a refractive prediction error within limits of ± 0.5 dioptre [5]. However, other mostly overlooked reasons for patient dissatisfaction are postoperative disparity of retinal image sizes between the two eyes of an individual or a meridional variation of retinal image size causing an image distortion [1, 2]. This can lead to headache, fusion problems, or in severe cases to a loss of stereopsis. From the literature, we know that aniseikonia is mostly below 0.5% in untreated eyes. An image size disparity of up to 2% is well tolerated by most patients, but retinal image size differences of 3% or more are sufficient to cause rapid fatigue [2, 6]. The tolerance of meridional retinal image sizes in terms of (monocular) image distortion or comparing both eyes of an individual has not yet been systematically investigated. Clinical measurement of retinal image size disparity is challenging and mostly unreliable and not part of routine clinical measurements [7]. Cohort studies or case reports which deal with aniseikonia are therefore rare. There are existing computer-based test strategies for aniseikona [3, 8] or classical test strategies [9, 10].

During ocular biometry prior to cataract surgery, all relevant data required for predicting the ocular magnification of both eyes are available [5]. Based on a schematic pseudophakic model eye, explicitly or implicitly defined by most of the (so-called theoretical-optical) IOL power calculation formulae, a number of parameters are obligatory to all calculation strategies—including axial length (AL) data, corneal front surface curvature data (radius in the flat meridian R1 in mm at flat axis Ra in ° and radius in the steep meridian R2 in mm at steep axis perpendicular to Ra), and the prediction of the effective lens position (ELP in mm). In addition, some formulae require more input data to specify the pseudophakic model eye such as the phakic anterior chamber depth (ACD in mm), the central thickness of the crystalline lens (LT in mm), or corneal back surface curvature data and central corneal thickness. The refractive indices of the aqueous (n_A_) and vitreous humour (n_V_) are typically derived from any classical schematic model eye, and the refractive indices of the cornea (n_C_) or the IOL (n_IOL_) are not required if the cornea and IOL are simplified using a thin lens model. In addition, the target refraction (TR in dpt) refers to the intended postoperative refraction at the spectacle plane.

Using the pseudophakic model eye underlying the IOL power calculation formula provides a simple and straight-forward option for estimating OOM [11, 12] and MOM [4, 13]. Using linear Gaussian optics (restricted to the paraxial space), OM can be derived in the pseudophakic eye for the spectacle-corrected eye (with any TR as correction), for the uncorrected eye (having a blurry image for any TR), or for the eye fully corrected by the IOL. For the simple case of rotationally symmetric refractive surfaces, a 2 × 2 matrix notation can be used for calculation [11], but for the more general case in which at least one surface in the eye is spherocylindric, a 4 × 4 matrix notation must be used [13].

The purpose of the present study was.

- to develop and present a concept for predicting the overall and meridional ocular magnification of an eye in the post-cataract situation based on ocular biometry and linear Gaussian optics using 4 × 4 matrix algebra;
- to predict the overall and meridional ocular magnification for both eyes of a patient:

- for the situation of a toric intraocular lens fully correcting the eye for the intended target refraction,
- and derive residual refraction for the situation of the respective non-toric intraocular lens (equivalent lens),
- for the situation of an equivalent lens with a spectacle correction of the residual cylinder;

- and to compare overall and meridional ocular magnification between both eyes of an individual based on a vector decomposition

using a large dataset from a cataract population measured with the IOLMaster 700 optical biometer.

## Methods

### Dataset for our analysis

For this retrospective study, we used a dataset containing a total of 32,198 biometrical measurements made with the IOLMaster 700 (Carl-Zeiss-Meditec, Jena, Germany) from two clinical centres (Augenklinik Castrop, Castrop-Rauxel, Germany, and Department of Ophthalmology, Johannes Kepler University Linz, Austria). All measurements were performed in a cataractous population, excluding pseudophakic eyes. Duplicate measurements of eyes, eyes in pharmacologically stimulated mydriasis (pupil width more than 5.2 mm), and incomplete records in the dataset were discarded. Measurement data indexed as being after refractive surgery, or having ectatic corneal diseases or other corneal pathologies were omitted from the dataset. The data were exported to a.csv data table using the data backup module of the IOLMaster 700 software. Data tables were reduced to the relevant parameters required for our data analysis, consisting of laterality (left or right eye), patient’s date of birth and examination date of the eyes, curvature of the corneal front surface (flat meridian: R1 at Ra; steep meridian: R2 perpendicular to Ra), ACD measured from the corneal front apex to the crystalline lens front apex in mm, and LT. The data were transferred to Matlab (Matlab version 2019b, MathWorks, Natick, USA) for further processing. The local ethics committee provided a waiver for this study (Ärztekammer des Saarlandes, 157/21).

### Preprocessing of the data

Custom software for data processing and analysis was written in Matlab. From the entire dataset, we selected patients with bilateral measurements taken on the same examination day, with all other examinations being discarded. Each patient’s age (age in years) was derived from their date of birth and the examination date. Without loss of generality, target refraction was set to zero (emmetropisation), the refractive indices of aqueous and vitreous humour were set to n_A_ = n_V_ = 1.336 (for the Castrop formula, the refractive index of the cornea was set to n_C_ = 1.376), and the back vertex distance for the spectacle correction was set to 12 mm. For comparison of both eyes of an individual, the dataset was split into right eyes (OD) and left eyes (OS).

### Toric intraocular lens power calculation and prediction of ocular magnification

Three different vergence-based formulae were used for calculating the intraocular lens power: the HofferQ formula [14], the Haigis formula [15], and the Castrop formula [16]. The HofferQ formula and the Haigis formula are based on a pseudophakic schematic model eye with 3 refracting surfaces (TR at spectacle plane, cornea as thin lens, and IOL as thin lens). In contrast to the HofferQ and Haigis formulae, the Castrop formula uses a pseudophakic schematic model eye with 4 refractive surfaces (TR at spectacle plane, cornea as a thick lens with front and back surface, and IOL as a thin lens). According to the formula definitions, the corneal power in both corneal meridians was calculated from R1 and R2 using the respective keratometer index (1.3375 and 1.3315 for the HofferQ and Haigis formulae) or for the corneal front and back surface using the refractive index of the cornea and aqueous humour for the Castrop formula. The formula constants were extracted from the IOLCon WEB site (https://iolcon.org, accessed on 20.03.2022) for the Tecnis lens (Johnson & Johnson, Brunswick, USA).

Lens power calculation, derivation of residual refraction, and extraction of OM for the corrected or uncorrected pseudophakic eye were performed using matrix algebra for toric optical systems [4, 13]. In general, the 4 × 4 power matrix *P* and the 4 × 4 translation matrix *T* are defined as:

$$\begin{array}{c}P=\left[\begin{array}{cc}U& P2\\ Z& U\end{array}\right]\\ T=\left[\begin{array}{cc}U& Z\\ T2& U\end{array}\right]\end{array}$$

where *U* refers to the 2 × 2 unity matrix, *Z* to the 2 × 2 zero matrix, and the 2 × 2 matrices *P*2 and *T*2 defined by

$$\begin{array}{c}P2=\begin{bmatrix}Pf+\left(Ps-Pf\right)\cdot\cos^{2}{\left(Pa\right)}&\left(Ps-Pf\right)\cdot\mathrm{si}n\left(Pa\right)\cdot\mathrm{co}s\left(Pa\right)\\\left(Ps-Pf\right)\cdot\sin\left(Pa\right)\cdot\cos\left(Pa\right)&Pf+\left(Ps-Pf\right)\cdot\sin^{2}{\left(Pa\right)}\end{bmatrix}\\T2=\begin{bmatrix}\frac dn&0\\0&\frac dn\end{bmatrix}\end{array}$$

*Pf*, *Ps*, and *Pa* describe the power in the flat and steep meridian and the axis of the flat meridian of a spherocylindric refractive surface, and *d* and *n* describe the geometric distance between subsequent surfaces and the refractive index of the optical medium [11]. The system matrix *S*, defined as the product of all power and translation matrices from object to image in reversed order, describes the properties of the entire optical system. With the 4 × 4 system matrix *S*, the slope ($$\alpha =\left[\begin{array}{c}{\alpha }_{x}\\ {\alpha }_{y}\end{array}\right]$$ in *X* and *Y*) and height ($$h=\left[\begin{array}{c}{h}_{x}\\ {h}_{y}\end{array}\right]$$ in *X* and *Y*) of the exiting ray are described by the respective slope and the height of the incident ray (*α*_0_ and *h*_0_) by:

$$\begin{bmatrix}\alpha\\h\end{bmatrix}=S\cdot\begin{bmatrix}\alpha_0\\h_0\end{bmatrix}$$

For a model with 3 refracting surfaces (pseudophakic model for the HofferQ or the Haigis formula) and objects at infinity, the system matrix reads:

$$S=\begin{bmatrix}SA&SB\\SC&SD\end{bmatrix}{=T}_{\mathrm V}\cdot P_{\mathrm{IOL}}\cdot T_{\mathrm{ELP}}\cdot P_{\mathrm C}\cdot T_{\mathrm{VD}}\cdot P_{\mathrm{TR}}$$

where P_IOL_, P_C_, P_TR_, T_V_, T_ELP_, and T_VD_ refer to the power matrices for the IOL, the cornea, the target refraction and to the translation matrices for the vitreous, the pseudophakic effective lens position, and the back vertex distance (which for this study was set to 12 mm without loss of generality).

For a model with 4 refracting surfaces (pseudophakic model for the Castrop formula) and objects at infinity, the system matrix reads:

$$S=\begin{bmatrix}SA&SB\\SC&SD\end{bmatrix}=T_{\mathrm V}\cdot P_{\mathrm{IOL}}\cdot T_{\mathrm{ELP}-\mathrm{CCT}}\cdot P_{\mathrm{CP}}\cdot T_{\mathrm{CCT}}\cdot P_{\mathrm{CA}}\cdot T_{\mathrm{VD}}\cdot P_{\mathrm{TR}}$$

where P_IOL_, P_CP_, P_CA_, P_TR_, T_V_, T_ELP-CCT_, T_CCT_, and T_VD_ refer to the power matrices for the IOL, the posterior and anterior corneal surfaces, the target refraction and to the translation matrices for the vitreous humour, the pseudophakic aqueous depth (effective lens position minus central corneal thickness (CCT)), the CCT, and the vertex distance. For simplification, CCT was set to 550 µm and instead of the measured corneal back surface curvature, the respective front surface curvature data scaled with a fixed ratio of 0.84 (0.84·R1 and 0.84·R2 for the flat and steep meridian) were used.

For calculation of the toric lens implant, we consider $S=T_{\mathrm V}\cdot P_{\mathrm{IOL}}\cdot S_{\mathrm{SUB}}$ with the subsystem matrix *S*_SUB_ defined by $S_{\mathrm{SUB}}=T_{\mathrm{ELP}}\cdot P_{\mathrm C}\cdot T_{\mathrm{VD}}\cdot P_{\mathrm{TR}}$ for the HofferQ and the Haigis formulae or ${S_{\mathrm{SUB}}=T}_{\mathrm{ELP}-\mathrm{CCT}}\cdot P_{\mathrm{CP}}\cdot T_{\mathrm{CCT}}\cdot P_{\mathrm{CA}}\cdot T_{\mathrm{VD}}\cdot P_{\mathrm{TR}}$ for the Castrop formula. For the corrected optical model, the lower right 2 × 2 matrix of *S* (SD) must be zero:

$$S=\left[\begin{array}{cc}SA& SB\\ SC& SD\end{array}\right]\equiv \left[\begin{array}{cc}.& .\\ .& Z\end{array}\right]$$

and after a short formula conversion, the upper right 2 × 2 matrix of P_IOL_ reads:

$${P2}_{\mathrm{IOL}}={T2}_{\mathrm V}^{-1}\cdot\left(\left(-{T2}_{\mathrm V}\cdot S_{\mathrm{SUB}}B\right)\cdot{S_{\mathrm{SUB}}B}^{-1}-U\right)$$

The 2 cardinal meridians (flat meridian P_IOL_f with axis P_IOL_a and steep meridian P_IOL_s) are extracted from P2_IOL_ using an eigenvalue decomposition. The equivalent power P_IOL_eq and the cylindric power P_IOL_cyl of the toric IOL implant are given by P_IOL_eq = 0.5·(P_IOL_f + P_IOL_s) and PIOLcyl = P_IOL_s-P_IOL_f.

In the next step, the IOL with the equivalent power P_IOL_eq is inserted and the residual (cylindric) refraction at the spectacle plane derived. The system matrix *S* is reformulated to:

$$S=S_{\mathrm{SUB}}\cdot P_{\mathrm{REF}}$$

with the subsystem matrix *S*_SUB_ defined by $S_{\mathrm{SUB}}=T_{\mathrm V}\cdot P_{\mathrm{IOL}}\cdot T_{\mathrm{ELP}}\cdot P_{\mathrm C}\cdot T_{\mathrm{VD}}$ for the HofferQ and the Haigis formulae or $S_{\mathrm{SUB}}=T_{\mathrm V}\cdot P_{\mathrm{IOL}}\cdot T_{\mathrm{ELP}-\mathrm{CCT}}\cdot P_{\mathrm{CP}}\cdot T_{\mathrm{CCT}}\cdot P_{\mathrm{CA}}\cdot T_{\mathrm{VD}}$ for the Castrop formula, and the power matrix P_REF_ describing the residual refraction at the spectacle plane. As the entire system is fully corrected with the (cylindric) spectacles, the lower right 2 × 2 matrix of *S* (SD) must be zero (*Z*). After a short formula conversion, we obtain that the upper right 2 × 2 matrix of P_TR_ (P2_REF_) reads:

$${P2}_{\mathrm{REF}}={S_{\mathrm{SUB}}C}^{-1}\cdot\left(-S_{\mathrm{SUB}}D\right)$$

Again, the 2 cardinal meridians (flat meridian P_REF_f with axis P_REF_a and steep meridian P_REF_s) are extracted from P2_REF_ using an eigenvalue decomposition. As the rotationally symmetric equivalent lens was considered, the spherical equivalent refraction is zero, and the cylindric refraction P_REF_cyl reads P_REF_cyl = P_REF_s − P_REF_f.

The 2 × 2 matrix M characterising OM is directly extracted from the 4 × 4 system matrix. In fully corrected systems, the lower right 2 × 2 matrix SD equals *Z*, and the OM is calculated from the lower left 2 × 2 matrix SC (M = SC). This is true for the situation where a fully correcting toric IOL is implanted or for the case where a rotationally symmetric IOL (e.g. with the equivalent power P_IOL_eq) is implanted and the residual refraction corrected at the spectacle plane. In situations where an IOL with its equivalent power is implanted and the residual refraction (refractive cylinder) remains uncorrected, both 2 × 2 matrices SC and SD are unequal to *Z*. This means that not all rays from the object passing through the optical system hit the same point in the image plane and the image will be blurred [12]. To extract OM for this blurry image, we e.g. identify the chief ray which passes through the pupil centre (assumed to be located within ACD behind the corneal front vertex). Expressed in matrix notation, we define the matrix characterising the subsystem from the object to the pupil plane as ($S_{\mathrm{PUP}}=T_{\mathrm{ELP}}\cdot P_{\mathrm C}\cdot T_{\mathrm{VD}}\cdot P_{\mathrm{TR}}$ for the HofferQ and the Haigis formulae or $S_{\mathrm{PUP}}=T_{\mathrm{ELP}-\mathrm{CCT}}\cdot P_{\mathrm{CP}}\cdot T_{\mathrm{CCT}}\cdot P_{\mathrm{CA}}\cdot T_{\mathrm{VD}}\cdot P_{\mathrm{TR}}$ for the Castrop formula) and postulate that

$${\begin{bmatrix}\propto\\h\end{bmatrix}=S}_{\mathrm{PUP}}\cdot\begin{bmatrix}\alpha_0\\h_0\end{bmatrix}\equiv\begin{bmatrix}.\\Z\end{bmatrix}$$

After some formula conversion, we obtain that the 2 × 2 matrix M characterising OM for the uncorrected optical system reads:

$$M=SC+SD\cdot\left(-{S_{\mathrm{PUP}}D}^{-1}\right)\cdot S_{\mathrm{PUP}}C$$

The meridional ocular magnification MOM in 2 cardinal meridians (magnification MOM1 in the magnification axis MOMa and magnification MOM2 in the magnification meridian) are extracted from M using eigenvalue decomposition. The mean overall ocular magnification OOM and the disparity between OM in the magnification meridian and the magnification axis DOM are calculated by OOM = 0.5·(MOM1 + MOM2) and DOM = (MOM2 − MOM1).

For calculating the difference between OM of both eyes, vector decomposition was performed to extract the components in the 0°/90° and in 45°/135° orientations. For symmetry reasons, the axis of all left eyes was mirrored at the vertical axis, meaning that the vector components in 45°/135° were flipped in sign [12]. Then the component for the right eyes was subtracted from the respective component for the left eye (ΔMEM_0°/90°_ = MEM_0°/90°_ (for left eyes) − MEM_0°/90°_ (for right eyes); and ΔMEM_45°/135°_ =  − MEM_0°/90°_ (for left eyes) − MEM_0°/90°_ (for right eyes).

### Statistics and linear prediction model for ocular magnification

The biometric data of the entire dataset, for right eyes and for left eyes, as well as the respective differences between left and right eyes, are shown descriptively with mean (MEAN), standard deviation (STD), median (MEDIAN), as well as the lower and upper boundaries of the 90% (CL90L and CL90U) confidence intervals. In an explorative analysis, the OM (OOM and DOM) is shown for scenario 1 with a fully correcting toric intraocular lens calculated for emmetropia, for scenario 2 with a non-toric equivalent lens and (sphero-)cylindric spectacle correction, as well as for scenario 3 with a non-toric equivalent lens without correction of the cylinder (blurred image). Data for the toric IOL (scenario 1) are provided in spherical equivalent power P_IOL_eq and cylinder power P_IOL_cyl, data for the residual refraction at the spectacle plane with implantation of the spherical equivalent lens (scenario 2) are given in cylinder power P_REF_cyl, and the ocular magnification for scenarios 1–3 is shown with overall ocular magnification OOM and with DOM values as the disparity in ocular magnification between the magnification meridian and magnification axis.

## Results

After quality approval of the dataset and filtering out incomplete data and patients with only one eye measured, a total of *N* = 9734 patients (measurements of 9734 right and 9734 left eyes, 5492 female and 4242 male patients, 5467 patients from Augenklinik Castrop and 4267 patients from Department of Ophthalmology, Johannes Kepler University Linz) were enrolled in our study. The mean age of the study population was 69 ± 15 years (median 73 years, 90% confidence interval from 43 to 85 years). Mean axial length was 23.68 ± 1.40 mm (confidence interval 21.84 to 26.14 mm). Table 1 shows the explorative data for the biometric parameters AL, CCT, ACD, LT, R1, and R2 for the entire dataset (*N* = 19,468 eyes), the dataset of OD and the dataset of OS, together with the difference between OS and OD (values shown are scaled by × 100).

**Table 1:** Explorative data of ocular biometry in the cataract population. The upper section refers to biometry of 19,468 eyes of 9734 patients. The second and third sections refer to the respective data after splitting into right (OD) and left (OS) eyes, with each patient contributing one eye to both the OD and OS groups. The last section shows the difference in biometric data between OS and OD eye (please note that all values in this section are scaled by × 100). AL, CCT, ACD, LT, R1, and R2 refer to axial length, central corneal thickness, anterior chamber depth, lens thickness, and radius of the corneal front surface in the flat and steep meridian. MEAN, STD, MEDIAN, and CL90L / CL90U refer to the mean, standard deviation, median, and lower / upper boundary of the 90% confidence interval

		AL in mm	CCT in mm	ACD in mm	LT in mm	R1 in mm	R2 in mm
All *N* = 19,468	MEAN	23.6769	0.5521	3.1324	4.6130	7.7927	7.6319
	STD	1.4015	0.0368	0.4171	0.4903	0.2795	0.2757
	MEDIAN	23.4866	0.5513	3.1257	4.6417	7.7837	7.6296
	CL90L / CL90U	21.8407 / 26.1416	0.4932 / 0.6137	2.4483 / 3.8437	3.6698 / 5.3409	7.3598 / 8.2594	7.1904 / 8.0842
OD *N* = 9734	MEAN	23.6971	0.5520	3.1339	4.6109	7.7962	7.6363
	STD	1.4068	0.0370	0.4168	0.4882	0.2788	0.2788
	MEDIAN	23.4998	0.5512	3.1270	4.6373	7.7873	7.6244
	CL90L / CL90U	21.8620 / 26.1562	0.4930 / 0.6138	2.4527 / 3.8471	3.6695 / 5.3408	7.3601 / 8.2650	7.1934 / 8.2650
OS *N* = 9734	MEAN	23.6567	0.5522	3.1310	4.6151	7.7892	7.6275
	STD	1.3960	0.0366	0.4179	0.4925	0.2801	0.2759
	MEDIAN	23.4746	0.5513	3.1237	4.6449	7.7803	7.6256
	CL90L / CL90U	21.8212 / 26.1190	0.4935 / 0.6136	2.4451 / 3.8420	3.6715 / 5.3411	7.3597 / 8.2539	7.1878 / 8.0794
OS − OD (× 100) *N* = 9734	MEAN	− 3.7162	0.0232	− 0.2910	0.4190	0.6943	− 0.8811
	STD	37.2622	1.1182	13.4435	20.5094	9.8423	9.7326
	MEDIAN	− 2.9992	0.0359	− 0.1850	0.2189	− 0.7290	− 0.7925
	CL90L / CL90U	− 49.5129 / 38.2883	− 1.3722 / 1.4356	− 21.3850 / 20.0738	− 29.4663 / 31.7477	− 14.4777 / 13.2308	− 14.7732 / 13.4130

**Fig. 2:**
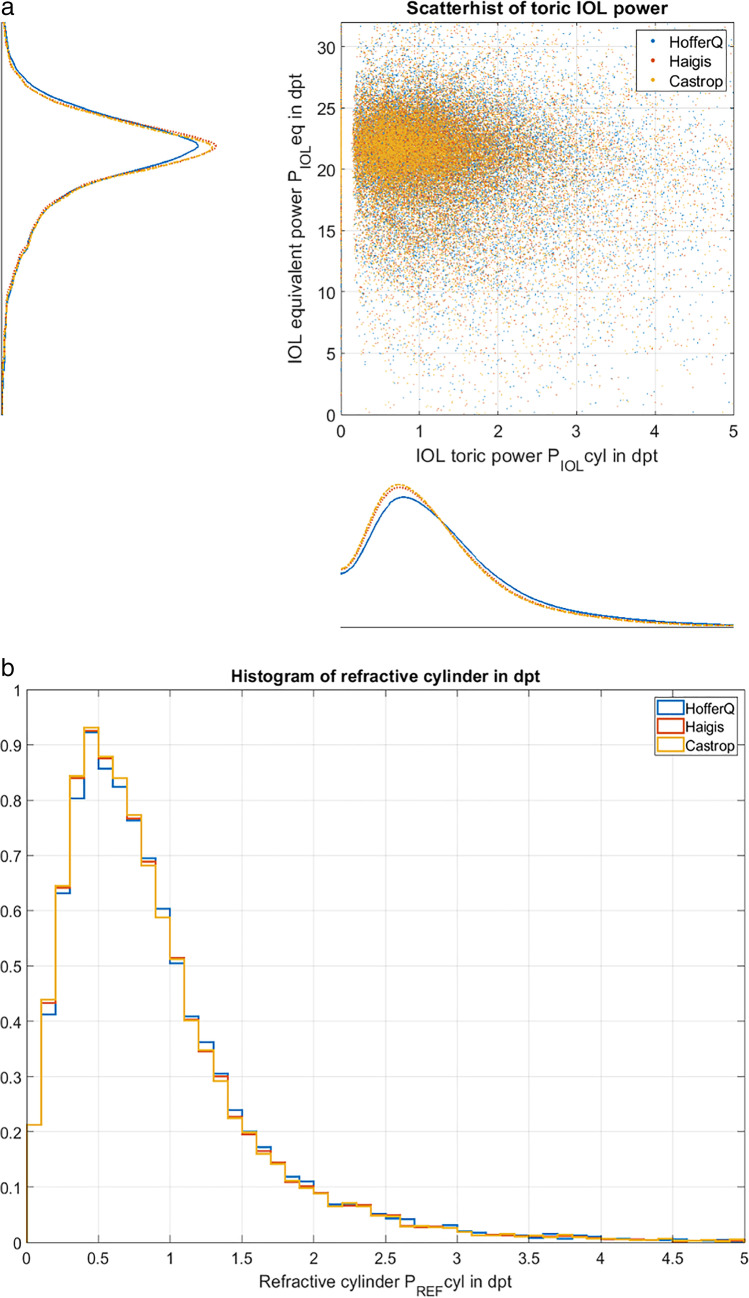
**a** Scatterhist (combined scatterplot and histogram) of the power of the toric lens for the entire dataset (*N* = 19,468, 9734 left and 9734 right eyes) calculated with the HofferQ, the Haigis, and the Castrop formulae. The equivalent power P_IOL_eq / cylindric power (P_IOL_cyl) is plotted on the *Y* / *X* axis of the scatterplot. The graph on the left indicates the kernel distribution for the equivalent power and the graph below the scatterplot the kernel distribution for the cylindric power. Please note that for small values of corneal astigmatism, the biometer does not provide measurements of corneal curvature separately for both meridians; therefore, the distribution for P_IOL_cyl does not show values close to zero. **b** Normalised histogram of the refractive cylinder P_REF_cyl of the predicted refraction at the spectacle plane calculated with the HofferQ, the Haigis, and the Castrop formulae where the non-toric equivalent lens with a power of P_IOL_eq is implanted. The data of the entire dataset (*N* = 19,468, 9734 left and 9734 right eyes) are included in this graph. Please note that for small values of corneal astigmatism, the biometer does not provide measurements of corneal curvature separately for both meridians; therefore, the distribution for P_REF_cyl does not show values close to zero

In Table 2, the explorative data for the refractive power of the toric intraocular lens derived with the HofferQ, the Haigis, and the Castrop formulae are displayed in terms of equivalent power P_IOL_eq and cylindric power P_IOL_cyl, together with the prediction of the cylindric residual refraction at the spectacle plane with the equivalent lens (P_IOL_eq) implanted instead of the toric lens. Data are shown for the entire dataset (*N* = 19,468 eyes) as well as separately for the dataset of OD and the dataset of OS (each *N* = 9734). Figure 2a provides the scatterhist for the power of the toric lens. On the *X* / Y axis of the scatterplot, the cylindric power P_IOL_cyl / equivalent power (P_IOL_eq) of the toric lens derived with the HofferQ, the Haigis, and the Castrop formulae respectively is provided. The graph on the left shows the kernel distribution for the equivalent power of the toric lens P_IOL_eq, and the graph below the scatterplot shows the kernel distribution for the cylindric power of the toric lens P_IOL_cyl. Figure 2b displays the normalised histogram for the predicted refractive cylinder where a non-toric intraocular lens with the equivalent power P_IOL_eq is implanted instead of a toric lens. As the biometer used for this study does not provide curvature data separately for the flat and steep corneal meridian for very small values of corneal astigmatism, the distributions of P_IOL_cyl in Fig. 2a and P_REF_cyl in Fig. 2b do not show values close to zero.

**Table 2:** Descriptive data of intraocular lens power in terms of equivalent (P_IOL_eq) and cylinder power (P_IOL_cyl) calculated for emmetropia together with the predicted (cylindrical) residual refraction at spectacle plane (P_REF_cyl) if the equivalent lens (P_IOL_eq) is implanted. The upper section refers to biometry of 19,468 eyes of 9734 patients. The second and third sections refer to the respective data after splitting into right (OD) and left (OS) eyes, with each patient contributing one eye to both the OD and OS group. Data are shown for the HofferQ, Haigis, and Castrop formulae. MEAN, STD, MEDIAN, CL90L, and CL90U refer to the mean, standard deviation, median, lower, and upper boundary of the 90% confidence interval

Data in dpt		HofferQ formula			Haigis formula			Castrop formula		
		P_IOL_eq	P_IOL_cyl	P_REF_cyl	P_IOL_eq	P_IOL_cyl	P_REF_cyl	P_IOL_eq	P_IOL_cyl	P_REF_cyl
All *N* = 19,468	MEAN	20.7715	1.3985	0.9152	20.7850	1.2993	0.8989	20.6730	1.2709	0.8955
	STD	4.4914	1.0998	0.7112	4.2269	1.0166	0.6986	4.3060	0.9905	0.6959
	MEDIAN	21.4304	1.1300	0.7441	21.4324	1.0524	0.7309	21.3111	1.0360	0.7280
	CL90L	12.5206	0.2706	0.1793	12.9926	0.2536	0.1761	12.6917	0.2479	0.1755
	CL90U	26.5880	3.4984	2.2785	26.2002	3.2515	2.2380	26.2607	3.1638	2.2294
OD *N* = 9734	MEAN	20.7312	1.3891	0.9090	20.7480	1.2907	0.8929	20.6313	1.2623	0.8894
	STD	4.4988	1.0878	0.7037	4.2367	1.0058	0.6912	4.3070	0.9796	0.6886
	MEDIAN	21.4108	1.1204	0.7391	21.4082	1.0438	0.7260	21.2868	1.0255	0.7232
	CL90L	12.5071	0.2682	0.1770	12.9538	0.2503	0.1739	12.6651	0.2434	0.1732
	CL90U	26.5011	3.4649	2.2575	26.1275	3.2066	2.2174	26.2000	3.1164	2.2090
OS *N* = 9734	MEAN	20.8117	1.4079	0.9213	20.8220	1.3079	0.9049	20.7147	1.2795	0.9015
	STD	4.4849	1.1116	0.7186	4.2191	1.0273	0.7059	4.3003	1.0012	0.7032
	MEDIAN	21.4556	1.1394	0.7497	21.4562	1.0622	0.7363	21.3430	1.0422	0.7336
	CL90L	12.5844	0.2722	0.1813	13.0350	0.2549	0.1781	12.7506	0.2510	0.1774
	CL90U	26.7257	3.5359	2.3106	26.2599	3.2864	2.2696	26.3315	3.2075	2.2603

Table 3 summarises the explorative data for OOM and DOM in scenario 1 (implantation of a fully correcting toric IOL), scenario 2 (implantation of a non-toric equivalent lens and spectacle correction of the residual cylindric refraction at spectacle plane), and scenario 3 (implantation of a non-toric equivalent lens without correction of the residual refraction) for the entire dataset and the subsets OD and OS. The magnification meridian / magnification axis in scenarios 1 and 3 is the steep / flat meridian of the cornea, whereas in scenario 2 the situation is reversed and the magnification meridian / magnification axis refers to the flat meridian / steep meridian of the cornea.

**Table 3:** Descriptive data of overall ocular magnification OOM and disparity in meridional ocular magnification between the magnification meridian and the magnification axis DOM based on calculations according to the HofferQ, Haigis, and Castrop formulae. Scenario 1 refers to a full correction of the eye for emmetropia using a toric lens. Scenario 2 / 3 refers to the situation of implantation of the respective non-toric equivalent lens and correction of the residual refractive cylinder with spectacles / the uncorrected eye with a blurry image. The upper section refers to biometry of 19,468 eyes of 9734 patients. The second and third sections refer to the respective data after splitting into right (OD) and left (OS) eyes, with each patient contributing one eye to both the OD and OS group. Data are shown for the HofferQ, Haigis, and Castrop formulae. MEAN, STD, MEDIAN, CL90L, and CL90U refer to the mean, standard deviation, median, lower, and upper boundary of the 90% confidence interval

OOM/DOM in mm/mrad (scaled by × 1000)		HofferQ formula			Haigis formula			Castrop formula		
		Scenario 1	Scenario 2	Scenario 3	Scenario 1	Scenario 2	Scenario 3	Scenario 1	Scenario 2	Scenario 3
All *N* = 19,468	MEAN	16.5459/ .0822	16.5463/ .1825	16.5468/ .0822	16.6276/ .0708	16.6279/ .1801	16.6276/ .0708	16.7005/ .0672	18.7008/ .1794	16.7005/ .0672
	STD	1.1887/ 0686	1.1888/ .1444	1.1887/ .0686	1.1562/ .0586	1.1563/ .1424	1.1562/ .0586	1.1761/ .0540	1.1762/ .1419	1.1761/ .0540
	MEDIAN	16.3621/ .0644	16.3624/ .1465	16.3621/ .0644	16.4455/ .0557	16.4456/ .1446	16.4455/ .0557	16.5182/ .0536	16.5184/ .1439	16.5182/ .0536
	CL90L	16.0667/ .0154	15.0669/ .0354	15.0667/ .0154	15.2059/ .0133	15.2061/ .0349	15.2059/ .0133	15.2344/ .0128	15.2354/ .0348	15.2344/ .0128
	CL90U	18.6176/ .2151	18.6183/ .4609	18.6176/ .2151	18.6488/ .1842	18.6494/ .4550	18.6488/ .1842	18.7733/ .1710	18.4766/ .4638	18.7733/ .1710
OD *N* = 9734	MEAN	16.5616/ .0818	16.5620/ .1814	16.5616/ .0818	16.6429/ .0705	16.6433/ .1791	16.6429/ .0705	16.7166/ .0668	16.7169/ .1783	16.7166/ .0668
	STD	1.1951/ .0681	1.1953/ .1413	1.1951/ .0681	1.1626/ .0581	1.1627/ .1411	1.1626/ .0581	1.1818/ .0535	1.1819/ .1406	1.1818/ .0535
	MEDIAN	16.3726/ .0152	16.3729/ .1458	16.3726/ .0641	16.4534/ .0554	16.4536/ .1439	16.4534/ .0554	16.5272/ .0532	16.5273/ .1434	16.5272/ .0532
	CL90L	15.0909/ .0152	15.0909/ .0348	15.0909/ .0152	15.2195/ .0132	15.2196/ .0343	15.2195/ .0132	15.2490/ .0126	15.2496/ .0342	15.2490/ .0126
	CL90U	18.6245/ .2123	18.6246/ .4563	18.6245/ .2123	18.6597/ .1823	18.6598/ .4497	18.6597/ .1823	18.7793/ .1690	18.7800/ .4476	18.7793/ .1690
OS *N* = 9734	MEAN	16.5302/ .0826	16.5306/ .01836	16.5302/ .0826	16.6122/ .0712	16.6125/ .1812	16.6122/ .0712	16.6844/ .0676	16.6847/ .1804	16.6844/ .0676
	STD	1.1820/ .0692	1.1821/ .1457	1.1820/ .0692	1.1496/ .0590	1.1497/ .1437	1.1496/ .0590	1.1702/ .0546	1.1703/ .1432	1.1702/ .0546
	MEDIAN	16.3519/ .0648	16.3522/ .1472	16.3519/ .0648	16.4377/ .0559	16.4378/ .1454	16.4377/ .0559	16.5097/ .0539	16.5099/ .1447	16.5097/ .0539
	CL90L	15.0546/ .0156	15.0549/ .0357	15.0546/ .0156	15.1869/ .0134	15.1879/ .0352	15.1869/ .0134	15.2182/ .0129	15.2183/ .0351	15.2182/ .0129
	CL90U	18.6142/ .2177	18.6146/ .4653	18.6142/ .2177	18.6431/ .1856	18.6441/ .4592	18.6431/ .1856	18.7654/ .1732	18.7656/ .4573	18.7654/ .1732

**Fig. 3:**
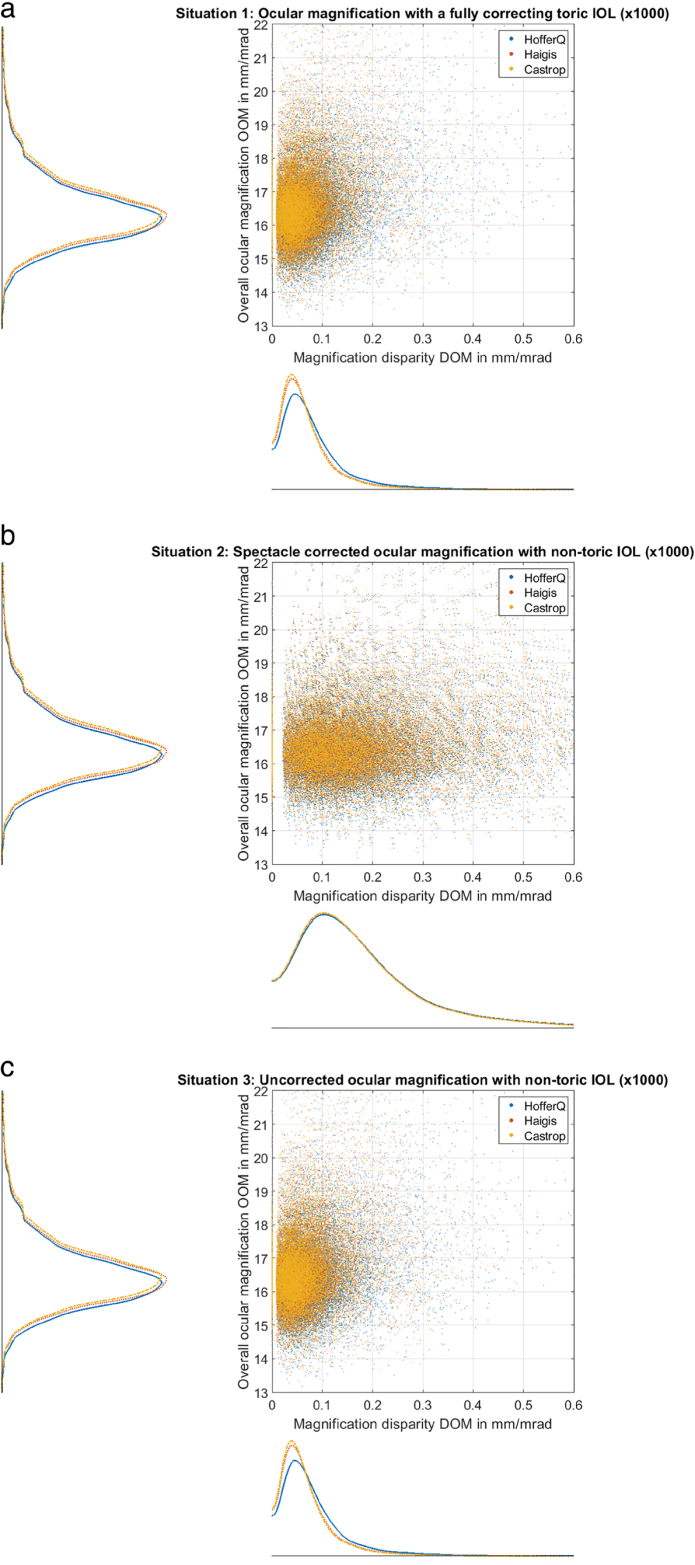
Scatterhist (combined scatterplot and histogram) of the disparity of ocular magnification (magnification meridian − magnification axis) of the 3 situations under test: *situation 1* (**a**) refers to a corneal astigmatism fully corrected by a toric lens implant for emmetropia, *situation 2* (**b**) */ 3* (**c**) refers to a non-toric lens implant calculated for emmetropic spherical equivalent refraction with correction of the residual cylinder at the spectacle plane / without correction of the residual cylinder (blurry image). All calculations are performed using a pseudophakic model eye according to the HofferQ, the Haigis, and the Castrop formulae. The data of the entire dataset (*N* = 19,468, 9734 left and 9734 right eyes) are included in these graphs. Please note that with situations 1 and 3, the magnification meridian refers to the steep corneal meridian whereas in situation 2 it refers to the flat corneal meridian

Figure 3 shows the overall ocular magnification OOM and the disparity of ocular magnification DOM based on calculations according to the pseudophakic model eyes used with the HofferQ, the Haigis, and the Castrop formulae. In situation 1 (upper graph, Fig. 3a), the corneal astigmatism is fully corrected by a toric lens implant, and the magnification meridian / magnification axis coincides with the steep corneal meridian. In situation 2 (middle graph, Fig. 3b), a non-toric equivalent lens is considered and the corneal astigmatism is fully corrected by a (cylindric) spectacle correction. In this situation, the magnification meridian / magnification axis coincides with the meridian where the spectacle correction shows its highest / lowest power (flat / steep corneal meridian). In situation 3 (lower graph, Fig. 3c), the same lens as in situation 2 is considered but the refractive cylinder remains uncorrected. In this situation, the magnification meridian / magnification axis again coincides with the steep / flat corneal meridian. In general, correction of the refractive cylinder with spectacles (situation 2) causes a systematically larger amount of DOM compared to a correction with a toric lens (situation 1) or corneal astigmatism which remains uncorrected (situation 3).

**Fig. 4:**
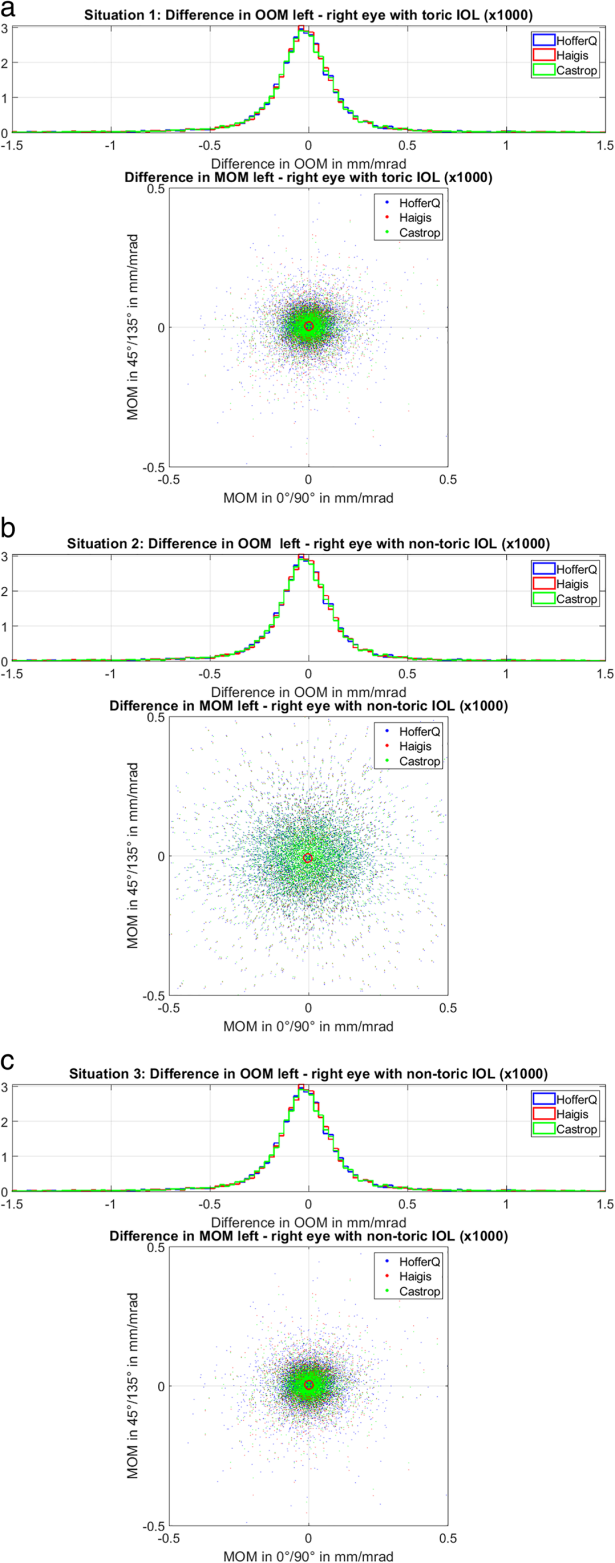
Intra-individual differences in ocular magnification between the left eye and the right eye of the 3 situations under test: *situation 1* (**a**) refers to a corneal astigmatism fully corrected by a toric lens implant for emmetropia, *situation 2* (**b**) */ 3* (**c**) refers to a non-toric lens implant calculated for emmetropic spherical equivalent refraction with correction of the residual cylinder at the spectacle plane / without correction of the residual cylinder (blurry image). The histograms show the difference in overall magnification OOM between both eyes, and the scatterplots display the differences in the vector components of MOM considered at 0°/90° meridians (*X*-axis) and 45°/135° meridians (*Y*-axis). Situation 2 shows a systematically larger scatter compared to situations 1 and 3 (the respective data for the scatter are listed in the text). All calculations are performed using a pseudophakic model eye according to the HofferQ, the Haigis, and the Castrop formulae. The marks in the scatterplots (blue x for HofferQ, red circle for Haigis, and green dot for Castrop) refer to the median centroids and indicate that no systematic differences between both eyes are observed. The data of the entire dataset (*N* = 19,468, 9734 left and 9734 right eyes) are included in these graphs

Figure 4 displays the intra-individual differences in ocular magnification between the left eye and the right eye of the 3 situations under test: the upper graph (situation 1, Fig. 4a) refers to a corneal astigmatism fully corrected by a toric lens implant for emmetropia, the middle graph (situation 2, Fig. 4b) to a non-toric lens implant calculated for emmetropic spherical equivalent refraction with correction of the residual cylinder at the spectacle plane, and the lower graph (situation 3, Fig. 4c) considers the same lens as in situation 2 but without correction of the residual cylinder (blurry image). The histograms show the difference in overall magnification OOM between both eyes, and the scatterplots display the differences in the vector components of MOM considered at 0°/90° meridians (*X*-axis) and 45°/135° meridians (*Y*-axis). Situation 2 shows a systematically larger scatter (SD of MOM *X*-axis / *Y*-axis with HofferQ: 0.1400/0.1228 e-3 mm/mrad, with Haigis: 0.1382/0.1211 e-3 mm/mrad, and with Castrop 0.1376/0.1206 e-3 mm/mrad) compared to situations 1 (SD of MOM *X*-axis / *Y*-axis with HofferQ: 0.0641/0.0565 e-3 mm/mrad, with Haigis: 0.0549/0.0483 e-3 mm/mrad, and with Castrop 0.0517/0.0455 e-3 mm/mrad) and 3 (SD of MOM *X*-axis / *Y*-axis with HofferQ: 0.0640/0.0565 e-3 mm/mrad, with Haigis: 0.0549/0.0484 e-3 mm/mrad, and with Castrop 0.0516/0.0457 e-3 mm/mrad). The marks in the scatterplots refer to the median centroids and indicate that no systematic differences between the two eyes are observed. For situation 1, the marks are located at coordinates *X*/*Y* = 14/38·e-7 mm/mrad (blue x) for HofferQ, *X*/*Y* = 11/32·e-7 mm/mrad (red circle) for Haigis, and *X*/*Y* = 12/31·e-7 mm/mrad (green dot) for Castrop. The respective marks for situation 2 are at coordinates *X*/*Y* =  − 30/ − 85·e-7 mm/mrad for HofferQ, *X*/*Y* =  − 29/ − 84·e-7 mm/mrad for Haigis, and *X*/*Y* =  − 29/ − 84·e-7 mm/mrad for Castrop, and for situation 3 at coordinates *X*/*Y* = 13/38·e-7 mm/mrad for HofferQ, *X*/*Y* = 11/32·e-7 mm/mrad for Haigis, and *X*/*Y* = 12/31·e-7 mm/mrad for Castrop, respectively. Without considering the symmetry and flipping the sign of the vector components at 45°/135° for left eyes, the median centroids are located at *Y* = 67/57/54·e-7 mm/mrad with the HofferQ/Haigis/Castrop formula for situation 1, − 152/ − 149/ − 149·e-7 mm/mrad for situation 2, and *Y* = 67/57/54·e-7 mm/mrad for situation 3.

## Discussion

It is well accepted in ophthalmology that anisometropia in terms of a disparity of distances between both eyes of an individual or differences in curvatures or power of refractive surfaces could cause aniseikonia [1, 17, 18]. However, even where all data for predicting ocular magnification of the pseudophakic eye are available with biometry prior to cataract surgery, IOL power calculation software typically does not provide such predictions. Furthermore, ophthalmologists might be unaware that spherocylindric elements in the eye may cause image distortions in a way that a circle or square in the object plane would be imaged to an ellipse or rectangle/rhombus in the retinal image plane. In a paraxial simplification, in a fully corrected optical system, all rays starting from an object point and passing through the system end up at the corresponding image point irrespective of the optical pathway [11, 12]. This implies that one single element in the system matrix in the second row characterises ocular magnification whereas the second element equals zero. When considering objects at infinity, the object size is undefined and ocular magnification refers to the retinal image size subdivided by the incident ray angle in radians (lower left element in the system matrix). When dealing with objects at finite distances, magnification refers to the ratio of image size to object size and the lower right element in the system matrix yields ocular magnification. For optical systems with astigmatic surfaces, we have to generalise this calculation strategy to 4 × 4 matrices instead of 2 × 2 matrices, and the 4 × 4 matrix is subdivided into four 2 × 2 submatrices [4, 13]. These 2 × 2 submatrices have the same meaning in an astigmatic system as the matrix elements in a non-toric system, but include information on the behavior of both cardinal meridians and the respective orientations, which can be extracted from the 2 × 2 matrix using eigenvalue decomposition.

Image distortions in optical systems with astigmatic surfaces are per se monocular effects. However, if both eyes of an individual include astigmatic surfaces, the distortions may or may not match between eyes (in absolute value and/or in direction). This means that in the best case the distortions are aligned, and in the worst case the magnification meridians of both eyes are perpendicular to each other, potentially making fusion of both retinal images difficult. Currently, there are no reliable clinical data on the tolerance of meridional magnification disparities, and there is no device on the market able to measure image distortion at the retina [7, 9]. However, in cataract surgery, preoperative biometry makes possible the option of estimating the amount of overall magnification disparity as well as the meridional disparity for the postoperative situation in the (spectacle) corrected or the uncorrected pseudophakic eye. Our results indicate that different lens power calculation concepts based on different pseudophakic model eyes yield slightly different results in the toric IOL power, the residual refraction, and in the OOM and MOM. However, these differences are rather small, meaning that that prediction of OM could be performed directly with the calculation concept that we use in our routine setting for lens power calculation.

If we consider the situation of a single eye, the results of IOL power, residual refraction, or OOM and MOM can be presented without vector decomposition into the 0°/90° and 45°/135° meridians. If we reference to the principal corneal meridians and fully correct corneal astigmatism with a toric lens or spherocylindric spectacles, the magnification conditions are quite different. With a toric lens the DOM is much lower compared to a correction of corneal astigmatism with spectacles, and the magnification meridian is located at the steep corneal meridian compared to the flat corneal meridian with spectacle correction. If corneal astigmatism remains uncorrected, the magnification meridian of the blurry image also coincides with the steep corneal meridian. However, in the general case where astigmatic surfaces are not axially aligned (crossed cylinders) or where more than 2 astigmatic surfaces are considered, general statements about the amount or orientation of the magnification meridians are not possible. Nevertheless, the calculation scheme presented in this paper could be applied in general to corrected or uncorrected optical systems with arbitrary numbers of astigmatic surfaces with cylinder axes in random orientations.

Surprisingly, a large number of eyes in a cataractous population would benefit from a toric lens implantation. Referring to the data of toric power of the IOL shown in Table 2 or to scatterhist in Fig. 2a, there is a wide range of toric IOL power with a range mostly between 0 and 5 dpt and a median of around 1.0 to 1.1 dpt. We have to be aware that for small values of corneal astigmatism the IOLMaster 700 seems to provide identical corneal radii R1 and R2 for both corneal meridians instead of a steep and flat meridian both with orientations, and the respective distribution of the toric IOL power lacks of data for values close to zero. If corneal astigmatism remains uncorrected due to implantation of a non-toric equivalent IOL, we could expect a residual cylinder at the spectacle plane ranging mostly between 0 and 4 dpt and with a median value of around 0.72 to 0.74 dpt (see also Table 2 and Fig. 2b). If we extract the relative distortion (DOM/OOM in %) from the data shown in Table 3, we obtain for the entire dataset 0.4884 ± 0.3888% / 1.0907 ± 0.8442% / 0.4884 ± 0.3887% for scenarios 1 / 2 / 3 with the HofferQ formula, 0.4187 ± 0.3309% / 1.0714 ± 0.8292% / 0.4187 ± 0.3309% for the Haigis formula, and 0.3968 ± 0.3085% / 1.0622 ± 0.8222% / 0.3968 ± 0.3085% for the Castrop formula, respectively. The respective upper boundary of the 90% confidence interval is 1.2316% / 2.7087% / 1.2316% for the HofferQ, 1.0538% / 2.6606% / 1.0538% for the Haigis, and 0.9824% / 2.6381% / 0.9824% for the Castrop formulae. This means that 5% of eyes after cataract surgery end up with an image distortion of 2.6 to 2.7% or more when a non-toric IOL is implanted and the residual cylinder corrected with spectacles, in contrast to around 1% distortion if a fully correcting toric IOL is implanted.

Comparing OOM of the left and right eye as shown in the histograms of Fig. 4, we find that the differences are mostly within limits of ± 0.0005 mm/mrad, which corresponds to a relative magnification difference of around ± 3%. As we aim for emmetropia in all 3 scenarios, there is no noticeable difference between OOM of both eyes comparing scenarios 1, 2, and 3. However, considering the difference in MOM between left and right eyes, we see a small scatter around zero for scenarios 1 and 3, but a much larger scatter for scenario 2 where a non-toric IOL is used and the residual cylindrical refraction is corrected at the spectacle plane. The median centroids as marked in the scatterplots of Fig. 4 are all located close to zero, and the distributions of the data points quite similar in *X* and *Y* direction. This indicates that our assumption of symmetry of left and right eyes with respect to the vertical axis is justified. If we do not consider such symmetry and do not flip the sign of the MOM vector components in 45°/135° for left eyes, the location of the median centroid shows a much larger shift in the *Y* direction (data in the “Results” section).

There are some limitations to our study: first of all, we used linear Gaussian optics for lens power calculation and for calculation of ocular magnification. This means that the calculations are restricted to paraxial rays and small ray angles. Unlike in linear optics, using full aperture raytracing ocular magnification cannot be defined in general as a function of biometric measures, since it depends on the ray height and the incident ray angle. In addition, we have assumed that the prediction of the axial lens position, as implicitly provided by several theoretical-optical lens power calculation formulae, sufficiently reflects the true axial lens position after cataract surgery. As we know, the effective lens position is used in some formulae to compensate for measurement or interpretation errors of biometric measures (e.g. converting corneal curvature to corneal power using a keratometer index) and this may slightly bias the result of our magnification prediction. Most of the lens power calculation formulae work on the basis of simplified thin lens models for the cornea, the intraocular lens, or both. The calculation scheme presented in this paper could, in general, deal with thick lens models for the cornea (as shown with the Castrop formula) or for the intraocular lens, provided that the geometry data of the corneal back surface (including corneal thickness) or the design data of the intraocular lens (front and back surface curvature, central thickness, and refractive index) are available. And last but not least, we restricted the study to prediction of retinal image sizes or disparities in overall or meridional magnification in terms of a transfer from the object size to the retinal image size. However, several other parameters of image processing in the retina or the visual cortex may play a role for the subjective tolerance of image size disparities, and these have not been considered in our calculation strategy.

In conclusion, from a routine biometry measurement prior to cataract surgery, we have all relevant measures required to predict overall and meridional ocular magnification for the pseudophakic eye after cataract surgery. With a pseudophakic optical model which is implicitly or explicitly defined by most of the lens power calculation concepts, the overall and meridional magnification can be extracted using simple matrix algebra. From the overall magnification, we could derive postoperative aniseikonia as the difference in retinal image sizes of both eyes. From meridional magnification, we could extract image distortion monocularly and/or the differences between left and right eye using vector decomposition. A prediction of ocular magnification during lens power calculation during biometry prior to cataract surgery may help to avoid eikonic problems as with a selection of equivalent and toric lens power and planning the target refraction aniseikonia and image distortions could be controlled within limits.

## Declarations

The local ethics committee provided a waiver for this study (Ärztekammer des Saarlandes, 157/21). All data processed in this study were already anonymized at the source before they were transferred to us for processing. Any back-tracing of the identity is not possible; therefore, an informed consent of the patient was not necessary.
